# Clinicopathological characteristics and prognosis of medullary thyroid microcarcinoma: a tumor with a similar prognosis to macrocarcinoma

**DOI:** 10.1186/s40001-023-01534-4

**Published:** 2023-11-28

**Authors:** Xin Wu, Binglu Li, Chaoji Zheng

**Affiliations:** Department of General Surgery, Peking Union Medical College Hospital, Chinese Academy of Medical Sciences & Peking Union Medical College, No. 1 Shuaifuyuan, Dongcheng District, Beijing, 100730 China

**Keywords:** Medullary thyroid carcinoma, Microcarcinoma, Macrocarcinoma, Cumulative survival rate

## Abstract

**Background:**

Tumor size plays an important role in the staging and treatment of thyroid carcinoma. A tumor with a maximum diameter of 1 cm or less is referred to as microcarcinoma. It is unclear if the clinicopathological characteristics and prognosis of medullary thyroid microcarcinoma (≤ 1 cm; MTMC) and macrocarcinoma (> 1 cm) differ. The present study aims to clarify the clinical features and prognosis of patients with MTMC.

**Methods:**

The patients with medullary thyroid carcinoma underwent radical operation at our hospital between December 2000 and January 2022 were retrospectively studied. A database was established for this study. Patients with MTMC and macrocarcinoma were grouped for comparison. The clinicopathological characteristics of the two groups were compared by χ^2^ test, Fisher’s exact test, t-test, and Mann–Whitney U test. Cumulative survival rates were presented by the Kaplan–Meier curves and compared using the log-rank test.

**Results:**

A total of 198 patients were included. Of them, 56 and 142 with MTMC and macrocarcinoma, respectively. Few patients in the MTMC group had lateral lymph node metastasis. One hundred and seventy-eight (89.9%) patients were followed up, with a median follow-up period of 61 (35, 105) months. The disease-free survival rate was significantly higher in the MTMC group (log-rank test, p = 0.032); however, there was no significant difference in the overall survival rate between the two groups (log-rank test, p = 0.083).

**Conclusions:**

Patients with MTMC have a lower risk of lateral lymph node metastasis and better disease-free survival than those with macrocarcinoma. However, there was no significant difference in the overall survival rate of both groups. MTMC should be treated in the same manner as macrocarcinoma.

## Background

The incidence of thyroid cancer has been gradually increasing worldwide. Nearly 600,000 new cases of thyroid cancer are reported annually, ranking 11th among all cases of cancer and accounting for approximately 3% of them [1, 2]. Further, thyroid cancer results in more than 40,000 deaths per year, accounting for approximately 0.4% of all tumor-related deaths [1, 2]. According to the 5th edition of the World Health Organization Classification of Endocrine and Neuroendocrine Tumors, thyroid neoplasms can be classified as developmental abnormalities, follicular cell-derived neoplasms, thyroid C cell-derived carcinoma, mixed medullary and follicular cell-derived carcinomas, and several other unusual neoplasms that occur in the thyroid [3]. Papillary thyroid carcinoma is the main subtype of follicular cell-derived neoplasms, while C cell-derived tumor refer to medullary thyroid carcinoma (MTC). Compared with papillary thyroid carcinoma, MTC has shown no obvious increase in the incidence rate. It accounts for only 1%–2% of all thyroid cancers but 8.6% of thyroid cancer-related deaths [4, 5]. Surgery is the main treatment modality for both sporadic and hereditary MTC and involves total thyroidectomy and central lymph node dissection. Some patients also require lateral lymph node dissection. Further, indicators such as calcitonin and carcinoembryonic antigen (CEA) can be used to monitor the disease in patients with MTC. Although radioactive iodine treatment is ineffective for MTC, new therapies, such as tyrosine kinase inhibitors, are promising [6, 7].

According to the eighth edition of the Cancer Staging Manual by the American Joint Committee on Cancer, tumor size plays an important role in the T staging of thyroid carcinoma [8, 9]. Tumors with a maximum diameter ≤ 1 cm and limited to the thyroid gland are staged as T1a. Meanwhile, a tumor with a maximum diameter less than or equal to 1 cm is defined as microcarcinoma [10–12]. In papillary thyroid carcinoma, the clinical characteristics, treatment strategies, and prognosis of microcarcinoma (≤ 1 cm) and macrocarcinoma (> 1 cm) differ significantly. Hemithyroidectomy is a widely used surgical procedure in papillary thyroid microcarcinoma. However, it is unclear if the clinicopathological characteristics and prognosis of MTC microcarcinoma and macrocarcinoma also differ. There is a lack of effective clinical evidence to support the formulation of treatment plans and evaluation of prognosis for patients with medullary thyroid microcarcinoma (MTMC). It remains to be determined whether MTMC has unique clinical characteristics and requires special treatment. Therefore, in the present study, we summarized the data from a large tertiary hospital to clarify the clinical characteristics and prognosis of MTMC.

## Materials and methods

### Patients

Medical records of all patients who underwent thyroid surgery at our hospital between December 2000 and January 2022 were retrospectively analyzed. Patients who met the following criteria were included in this study: (1) MTC confirmed by postoperative paraffin pathology, (2) radical surgery, and (3) complete and accessible medical records. Patients with a history of head and neck radiation or other head and neck cancers were excluded. Patients who underwent only hemithyroidectomy were also excluded. This study was conducted using a retrospective database. General characteristics, symptoms, examination results, surgical information, pathological details, and follow-up information were recorded and analyzed. All the data were recorded and checked separately by two independent doctors. This study was reviewed and approved by the Institutional Review Board of Peking Union Medical College Hospital (I-23PJ1067). All patients provided written informed consent for the surgery. The requirement for informed consent for the publication of the data was waived owing to the retrospective nature of the study.

### Treatment

All included patients underwent thyroid function tests and ultrasonography. Computed tomography was performed for patients with obvious neck masses or airway compression. Surgery was performed under general anesthesia with the patient in the supine position with hypsokinesis of the head. All patients underwent total thyroidectomy and central compartment lymph node dissection. Only patients with abnormal lateral lymph nodes on preoperative ultrasonography underwent lateral compartment lymph node dissection. Neck drainage tubes were routinely placed during surgery. The patients started drinking water 6 h after surgery and started eating the next day. Euthyrox was prescribed as thyroid hormone replacement therapy to all patients. Follow-ups were conducted through outpatient interviews, telephone calls, e-mails, and WeChat. The first follow-up was performed 1 month after surgery and then every 3 months for 1 year. If the results were normal, the follow-up schedule was changed to once every 6 months. The follow-up examinations included thyroid function tests, ultrasonography, and computed tomography.

### Definition

In the present study, reference levels of calcitonin, CEA, free triiodothyronine, and free thyroxine were < 10 pg/mL, ≤ 5 ng/mL, 1.8–4.1 pg/mL, and 0.81–1.89 ng/dL, respectively. A bilateral tumor was defined as the presence of lesions in both the left and right thyroid lobes. Multifocal tumors were defined as at least two lesions in bilateral or unilateral lobes. Major tumor size was defined as the diameter of the largest lesion. Total tumor size was defined as the sum of the diameters of all lesions. The Clavien-Dindo system was used to define and classify postoperative complications [13]. Imaging abnormalities or significant elevations in calcitonin levels during follow-up were considered indicators of tumor recurrence, and pathological evidence was not required. Disease-free survival (DFS) and overall survival (OS) time were defined as the time from the date of surgery to the date of tumor recurrence and death, respectively. If the patient did not relapse or die, the cut-off point was the date of the last follow-up.

### Statistical analysis

The Statistical Package for Social Sciences software (version 25.0; IBM Corp., Armonk, NY, USA) was used for the statistical analysis. Categorical variables were presented as absolute numbers and frequencies and compared with χ^2^ test and Fisher’s exact test. Continuous variables with normal distribution were described as mean ± standard deviation and compared using the t-test, while those with skewed distribution were presented as median (25th, 75th) values and compared using the Mann–Whitney U test. Kaplan–Meier curves with log-rank tests were used to describe and analyze cumulative survival rates. Statistical significance was set at p < 0.05.

## Results

Based on the inclusion and exclusion criteria, a total of 198 patients were included in this study. The general information of all patients is presented in Table 1. All patients underwent radical surgery and 23 of them had complications. The detailed information of complications is presented in Table 2. Based on the postoperative pathological results, all patients were divided into two groups: micro- (tumor ≤ 1 cm) (n = 56) and macro- (tumor > 1 cm) (n = 142) MTC groups. The general characteristics and operative information of both groups are presented in Table 3. The pathological results of both groups are compared in Table 4.

**Table 1:** General information of all 198 patients with medullary thyroid carcinoma

Variables	Value
Sex (n,[%])	
Male	87 (43.9%)
Female	111 (56.1%)
Age (years)	47.4 ± 12.6
Symptom (n)	
Neck discomfort	15
Dysphagia	5
Hoarseness	4
Headache	4
Dyspnea	3
Diarrhea	3
Palpitations	3
Weakness	3
Asymptomatic	158
Classification (n,[%])	
Hereditary	32 (16.2%)
Sporadic	166 (83.8%)
Scope of surgery (n,[%])	
Total thyroidectomy with central and lateral lymph node dissection	110 (55.6%)
Total thyroidectomy with central lymph node dissection	88 (44.4%)

**Table 2:** Complications information of 23 patients with medullary thyroid carcinoma

Complications	Value (n)	Treatment	Clavien-Dindo classification
Hypocalcemia	5	Conservative treatment	II
Lymphatic leakage	3	Conservative treatment	I
Recurrent laryngeal nerve paralysis	3	Conservative treatment	II
Horner syndrome	2	Conservative treatment	II
Bleeding	2	Reoperation	IIIb
Pulmonary infection	2	Conservative treatment	II
Fever	2	Conservative treatment	I
Jugular vein thrombus	1	Conservative treatment	II
Wound infection	1	Conservative treatment	I
Pleural effusion	1	Computed tomography guided puncture drainage	IIIa
Pheochromocytoma crisis	1	Transfer to the intensive care unit	IVa

**Table 3:** Comparison of general characteristics and operative information between patients with micro- and macro- medullary thyroid carcinoma

	Micro (n = 56)	Macro (n = 142)	*p*
Male/female (n)	27/29	60/82	0.447
Age (years)	46.9 ± 12.1	47.7 ± 12.8	0.693
BMI (kg/m^2^)	23.3 ± 3.4	23.8 ± 3.5	0.390
Hereditary (n)	8	24	0.652
Hashimoto's disease (n)	9	24	0.888
Calcitonin ˃10 pg/mL (n)	32	94	0.233
CEA ˃5 ng/mL (n)	21	83	0.008
FT3 (pg/mL)	3.1 ± 0.4	3.1 ± 0.5	0.594
FT4 (ng/dL)	1.3 ± 1.0	1.3 ± 0.9	0.997
Sonographic features (n)			
Spiculated margin	41	87	0.113
Microcalcification	30	73	0.784
Hypervascularity	41	101	0.769
ASA ≥ III (n)	0	6	0.187
Operative time (min)	105 (80, 169)	135 (100, 180)	0.029
Postoperative complications (n)	5	18	0.459
Central LNM (n)	22	76	0.071
Lateral LND (n)	23	87	0.010
Lateral LNM (n)	16	66	0.021

*BMI* body mass index, *CEA* carcinoembryonic antigen, *FT3* free triiodothyronine, *FT4* free thyroxine, *ASA* American Society of Anesthesiologists, *LNM* lymph node metastasis, *LND* lymph node dissection

**Table 4:** Comparison of the pathological findings of patients with micro- and macro- medullary thyroid carcinoma

	Micro (n = 56)	Macro (n = 142)	*p*
Lobe (left/right) (n)	28/28	72/70	0.929
Bilateral tumor (n)	8	38	0.061
Multifocal tumor (n)	10	42	0.091
Major tumor size (cm)	0.8 ± 0.2	2.4 ± 1.2	< 0.001
Total tumor size (cm)	0.9 ± 0.4	2.8 ± 1.9	< 0.001
Capsular invasion (n)	12	45	0.151
Extrathyroidal invasion (n)	4	18	0.265
Parathyroid gland dissection (n)	19	47	0.911
No. of harvested central LN	6 (3, 11)	8 (4, 12)	0.121
No. of positive central LN	0 (0, 1)	1 (0, 4)	< 0.001
No. of harvested lateral LN	24 (15, 34)	21 (16, 32)	0.874
No. of positive lateral LN	2 (0, 4)	4 (1, 9)	0.057
N staging (n)			0.070
0	29	56	
1a	11	20	
1b	16	66	
TNM staging (n)			< 0.001
I	28	28	
II	1	23	
III	10	18	
IV	17	73	
TNM staging (n)			
I/II–IV	28/28	28/114	< 0.001

*LN* lymph node, *TNM* tumor, nodes and metastases

Compared with the micro group, the macro group had a higher CEA level, longer operation time, more patients with lateral lymph node metastasis, and more patients who underwent lateral compartment lymph node dissection. It is worth mentioning that the number of patients with central lymph node metastasis was not significantly different between the two groups. Patients in the macro group had more positive central lymph nodes and higher tumor, nodes and metastases (TNM) staging. However, there was no significant difference in the number of patients with bilateral tumors, multifocal tumors, capsular invasion, extrathyroidal invasion, and high-N staging between the two groups.

**Fig. 1:**
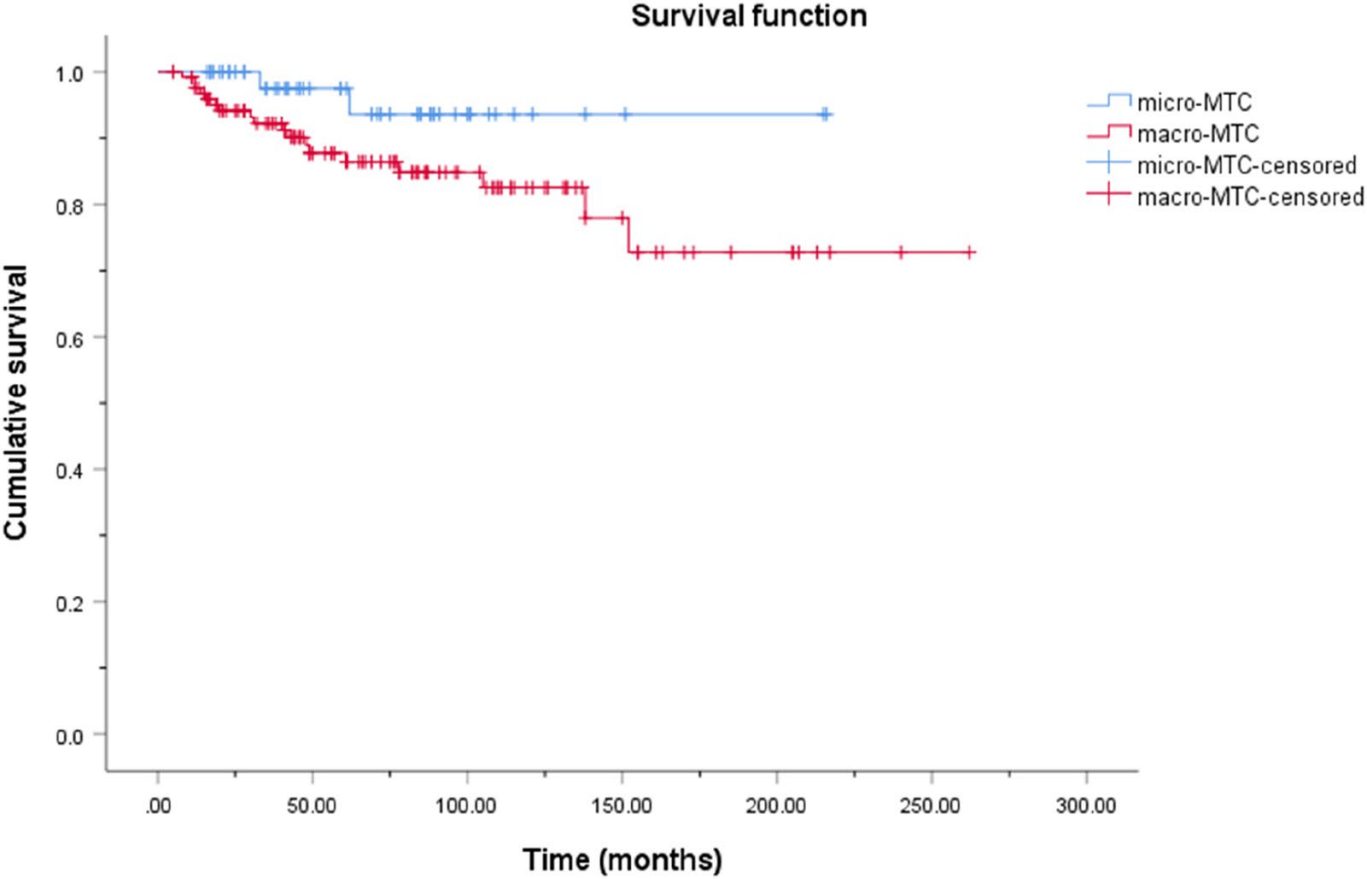
Kaplan–Meier cumulative overall survival curves for medullary thyroid microcarcinoma and macrocarcinoma

**Fig. 2:**
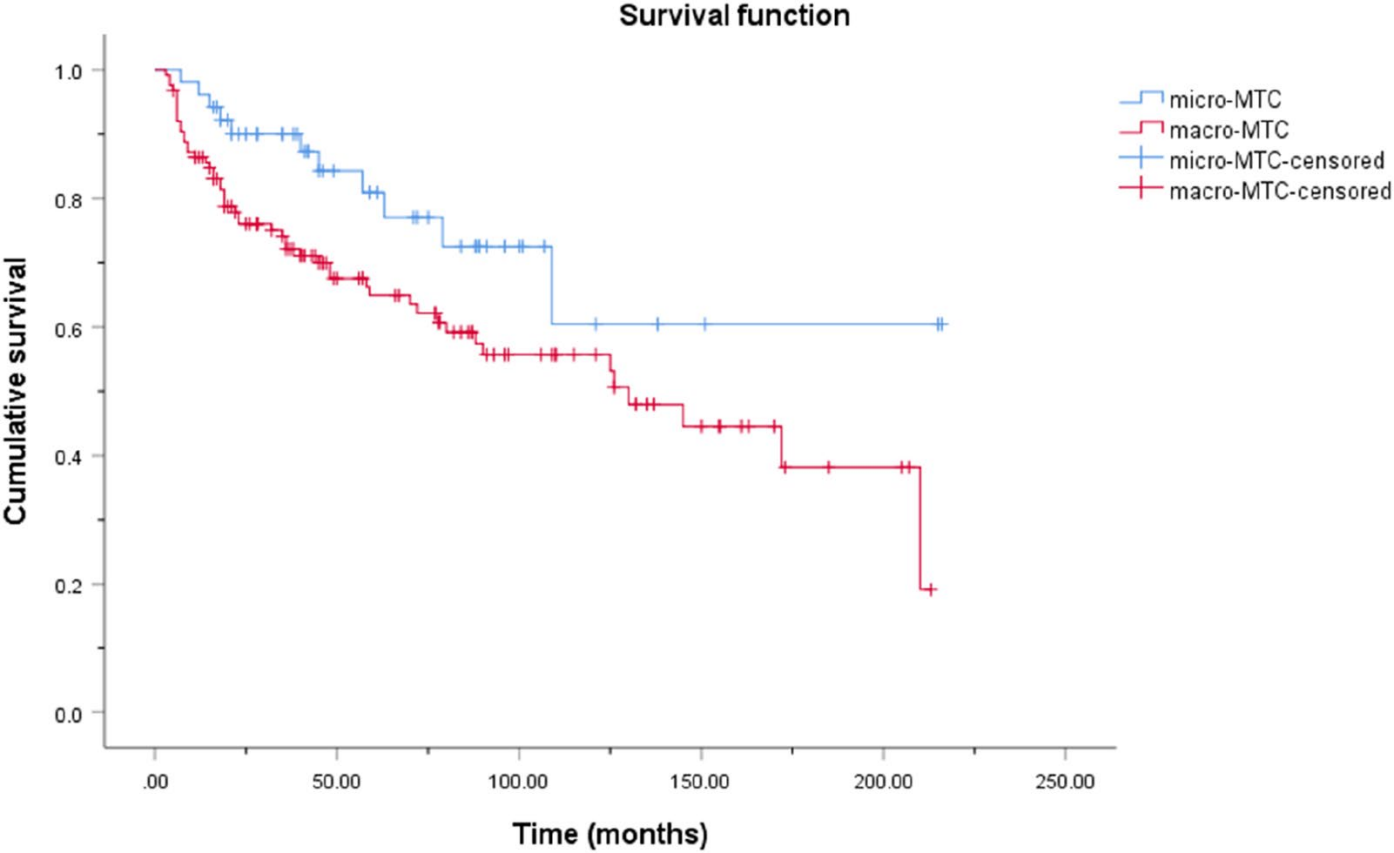
Kaplan–Meier cumulative disease-free survival curves for medullary thyroid microcarcinoma and macrocarcinoma

As of December 2022, 178 (89.9%) patients were followed up, with a median follow-up time of 61 (35, 105) months, and the other 20 (10.1%) patients were lost to follow-up. Of the patients who were followed up, 116 (65.2%) survived without tumors, 42 (23.6%) survived with tumors, and 20 (11.2%) died. The cumulative OS rates of the micro and macro groups are presented and compared in Fig. 1. The 5-, 10-, and 15-year cumulative OS rates of MTMC were 97.5%, 93.6%, and 93.6%, respectively. For macrocarcinoma, the rates were 87.7%, 82.5%, and 72.7%, respectively. There was no significant difference in the OS rate of the two groups (log rank, p = 0.083). The cumulative DFS rates of the micro and macro groups are presented and compared in Figs. 2. The 5-, 10-, and 15-year cumulative DFS rates of MTMC were 80.9%, 60.4%, and 60.4%, respectively. For macrocarcinoma, the rates were 64.9%, 55.7%, and 38.2%, respectively. The DFS rate was significantly better in the micro group (log rank, p = 0.032).

## Discussion

The present study summarizes the clinical characteristics and prognosis of patients with MTMC. We found that, although patients with MTMC had less lateral lymph node metastasis and better DFS, the local invasion, central lymph node metastasis, and OS rates were not superior to those in macrocarcinoma. Therefore, we suggest that the same treatment strategies and surgical approaches should be adopted for MTMC and macrocarcinoma.

Over the past decades, the incidence of thyroid carcinoma has increased by more than 300% [14]. Although the growth rate of MTC is not evident, the proportion of MTMC has increased [15]. Therefore, greater attention is being paid to this disease. Owing to the unique endocrine function of the thyroid gland, it is important to pay attention to the quality of life of patients during treatment. In selected papillary thyroid carcinomas, such as microcarcinoma, hemithyroidectomy may be as effective as total thyroidectomy. Whether similar management is applicable in MTC has also been investigated. Some early studies with small sample sizes had questioned whether the extent of radical surgery required for MTMC is same as that for macrocarcinoma [16–18]. Raffel et al. [16] retrospectively studied 15 patients with small sporadic MTC and concluded that total thyroidectomy and lymph node dissection were not mandatory for selected patients. Hamy et al. [17] performed a prospective multicenter study on 43 patients with sporadic MTMC. They found that lymph node metastasis was uncommon, and they questioned the significance of systematic central neck dissection in MTMC. However, the present study indicated that the invasiveness of MTMC was similar to that of macrocarcinoma. The two groups of tumors with different sizes were similar in terms of capsular invasion, extrathyroidal invasion, central lymph node metastasis, and N staging. This may be related to the strong invasiveness of MTC. Even if the tumor diameter is small, it is prone to local invasion and lymphatic metastasis.

The aggressive nature of MTMC has been further confirmed by several different studies. Kazaure et al. [15] used the Surveillance, Epidemiology, and End Results database to report on 310 patients with MTMC and found a 10-year OS rate of 91.6%. They recommended thyroidectomy and central compartment lymph node dissection for preoperatively diagnosed MTMC. Kim et al. [19] performed a meta-analysis of 15 studies and compared the clinical features of MTMC with those of macrocarcinoma. They observed that MTMC has aggressive features and accordingly suggested a similar treatment strategy for MTMC and macrocarcinoma. Li et al. [20] retrospectively compared the clinical and ultrasonographic characteristics of MTMC and papillary thyroid microcarcinoma and found that MTMC was more likely to have lymph node metastasis. Machens et al. [21] studied 233 patients with MTMC and found that lymph node metastases were common. In a recent study, Kesby et al. [22] studied 42 patients with MTMC, of which five (12%) had lymph node metastases; during a median follow-up of 6.6 years, five (12%) had recurrence, and three (7%) died. In summary, studies of MTMC have led to very different conclusions from those of papillary thyroid microcarcinoma.

Indications for lymph node dissection in patients with MTMC have not clearly established [4]. There was even guideline that indicated central compartment lymph node dissection as unnecessary in MTMC [23]. Lymph node metastasis is associated with MTC prognosis. Regardless of the tumor size, the central and ipsilateral lymph node metastasis rates of MTC can be as high as 50% to 75% [4]. Therefore, although controversial, total thyroidectomy and central compartment lymph node dissection remain mainstream surgical procedures for MTC. In our study, there was no difference between MTMC and macrocarcinoma in terms of local invasion or central lymph node metastasis. This finding supports the aforementioned perspective. Extrathyroidal invasion can easily lead to lymph node metastasis [15, 24–26]. This may be the reason for similar central lymph node metastases in MTMC and macrocarcinoma. MTMC therefore requires the same extent of surgery as macrocarcinoma. A recent systematic review revealed that there remains a lack of evidence on surgical procedures less invasive than total thyroidectomy and lymph node dissection for MTMC [27]. In the present study, the operative time of MTMC was shorter than that of macrocarcinoma. Because there were more patients underwent lateral lymph node dissection in the macro group. However, the postoperative complication rates between micro and macro groups were similar. It indicated that as long as the surgery was performed with precision, even lateral lymph node dissection would not significantly increase the incidence of complications.

Although controversial, the management approaches for papillary thyroid microcarcinoma are becoming more conservative [10]. The indolent biological nature of microcarcinoma may not have a significant effect on patient prognosis. However, it is unclear whether a similar strategy can be applied to MTMC and whether MTMC and macrocarcinoma should be treated differently. A meta-analysis showed that the DFS rate of MTMC was better than that of macrocarcinoma [19], which is consistent with the present findings. However, no significant difference was observed in the OS or local invasion between both lesions. According to our data, there were fewer lateral metastases in MTMC, which may be the reason for the better DFS of MTMC. Because lymph node metastasis is an important cause of tumor recurrence. On the other hand, the OS may be more closely related to the invasiveness of the tumor. This could explain the lack of a significant difference in the OS rates of the two groups. For this reason, it is necessary to implement the same treatment strategies for MTMC and macrocarcinoma.

This study had some limitations. First, owing to its retrospective nature, registration information and patient volume could not be planned beforehand. Second, the sample size of this single-center study was limited, with a low incidence of MTC. Third, 20 patients (10.1%) who were lost to follow-up had a high possibility of death, which may have affected the results on patient prognosis. In future, multicenter, prospective, controlled clinical trials should be performed to obtain more robust data.

## Conclusions

The present study summarized the clinicopathological characteristics and prognosis of MTMC and compared them with those of macrocarcinoma. MTMC showed local invasion, central lymph node metastasis, and OS rates similar to those of macrocarcinoma; however, MTMC had better lateral lymph node metastasis and DFS rates. Based on the data from this study, the same treatment strategies are recommended for MTMC and macrocarcinoma.

## Data Availability

Research data are available from the corresponding author on reasonable request.

## Abbreviations

MTC: Medullary thyroid carcinoma
CEA: Carcinoembryonic antigen
MTMC: Medullary thyroid microcarcinoma
DFS: Disease-free survival
OS: Overall survival
TNM: Tumor, nodes and metastases
